# Gastric Cancer Presenting as Persistent Pneumonia: An Unusual Case Report

**DOI:** 10.7759/cureus.14024

**Published:** 2021-03-21

**Authors:** Aretha Kou, Jinal K Patel, Itioye Adetula, Johnathan Frunzi

**Affiliations:** 1 Internal Medicine, Medical Center of Trinity, Trinity, USA

**Keywords:** gastric cancer, fifth most common cancer, male sex, alcohol use, obesity, helicobacter pylori, dysphagia, abdominal pain, nausea, smoking

## Abstract

Worldwide, gastric cancer is the fifth most common cancer and the third leading cause of cancer deaths, which carries a poor prognosis as only 28.3% are expected to survive after five years. The incidence varies depending on the geographical locations and dietary patterns. Here, we present a case of a 59-year-old Hispanic male with a 10-month history of recurrent bilateral pneumonia and dysphagia. Diagnostic workup revealed metastatic gastric adenocarcinoma.

## Introduction

Worldwide, gastric cancer is the fifth most common cancer and the third leading cause of cancer deaths [1-3]. Gastric cancer carries a poor prognosis as only 28.3% are expected to survive after five years [1-5]. The incidence of gastric cancer is less common in North America, Australia, New Zealand, Southern Asia, and North and East Africa [1]. However, there is a higher incidence in Eastern Asia, Central, Eastern Europe, and South America [2]. The higher incidence is attributed to dietary patterns, socioeconomic status, and Heliobacter pylori infections [2]. Despite the highest incidence in Eastern Asia, Central and Eastern Europe, and South America, it is more common among Hispanics, African Americans, and Native Americans when compared to non-Hispanic whites in the United States [2]. Also, gastric cancer is two-three times more common in men than in women [1]. 

On the other hand, there is a lower incidence of gastric cancer in the United States [2]. Despite the number of new cases declining by 1.5% annually in the United States for the past 10 years [6], The American Cancer Society estimated 27,600 new cases of gastric cancer in 2020 [6]. Of that, 11,010 people were estimated to die [6]. In the United States, most cases of gastric cancer by the time of diagnosis have progressed to advanced, incurable disease [1,3,4]. This further illustrates the need to screen patients with risk factors (smoking, male gender, ethnicity, diet, obesity, Helicobacter pylori [H. Pylori], alcohol, abdominal radiation therapy) [1-5,7]. Here, we present a case of a 59-year-old Hispanic male with a 10-month history of recurrent bilateral pneumonia and dysphagia diagnosed as gastric cancer.

## Case presentation

A 59-year-old Hispanic male with a past medical history of diabetes, hyperlipidemia, chronic obstructive pulmonary disease (COPD), and remote smoking history (quit 29 years ago) was admitted to the hospital by his pulmonologist for recurrent bilateral pneumonia over the past 10 months. The patient's initial symptom was a cough associated with lying supine, eating, or drinking. He also reported that his oxygen saturation would decrease to the 80s while sleeping and unintentional weight loss of 40 pounds over the past 10 months. On further questioning, the patient reported having progressive dysphagia from solids to liquid over the past 10 months.

He reported being admitted to a different hospital approximately three-weeks prior, where he was diagnosed with persistent pneumonia and treated with antibiotics and steroids. The patient's hospital course was complicated secondary to pleural effusion, which required a thoracentesis. Pleural fluid analysis was negative for malignancy. CT scan of the chest during his previous hospital stay revealed extensive bilateral infiltrates and a 3 cm density in the left upper lobe. He was discharged and to follow up as outpatient with his pulmonologist.

**Figure 1:**
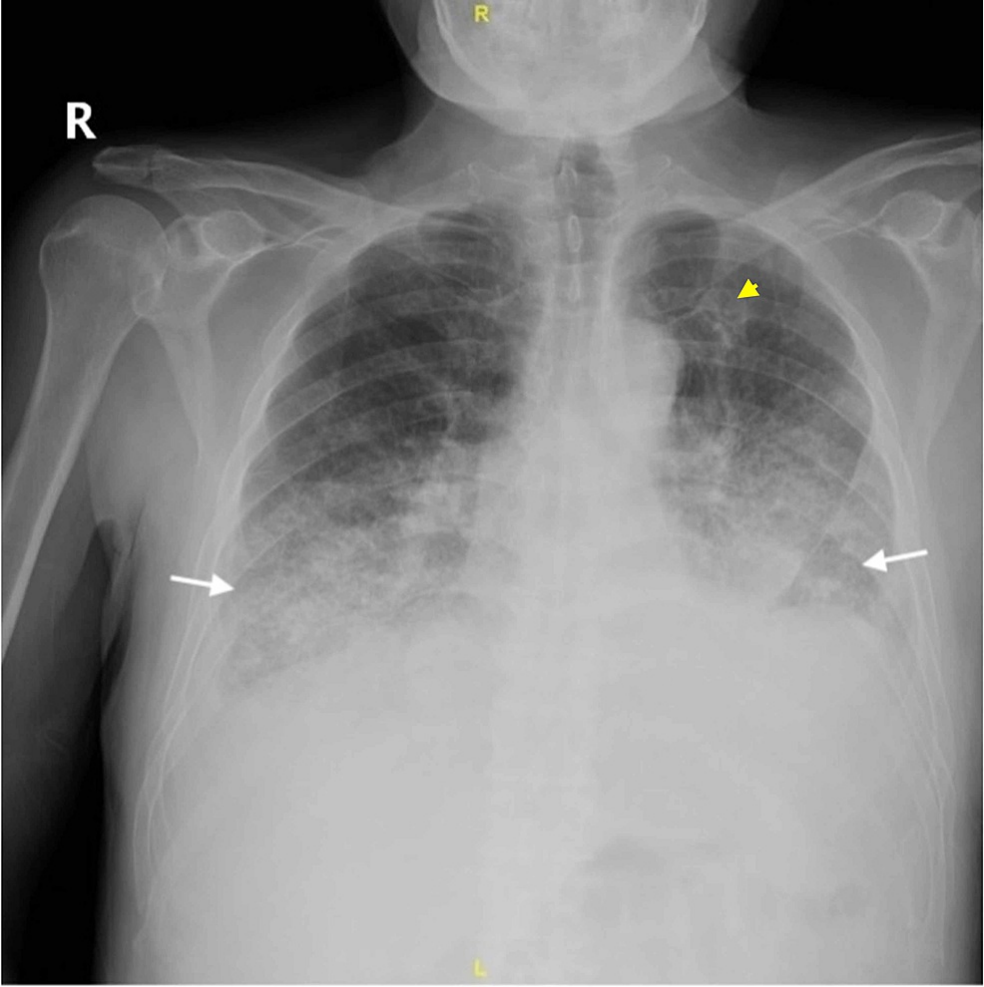
Chest X-ray. Bilateral lower lobe effusion and consolidation (lower arrows), left upper lobe nodule (yellow arrow).

**Figure 2:**
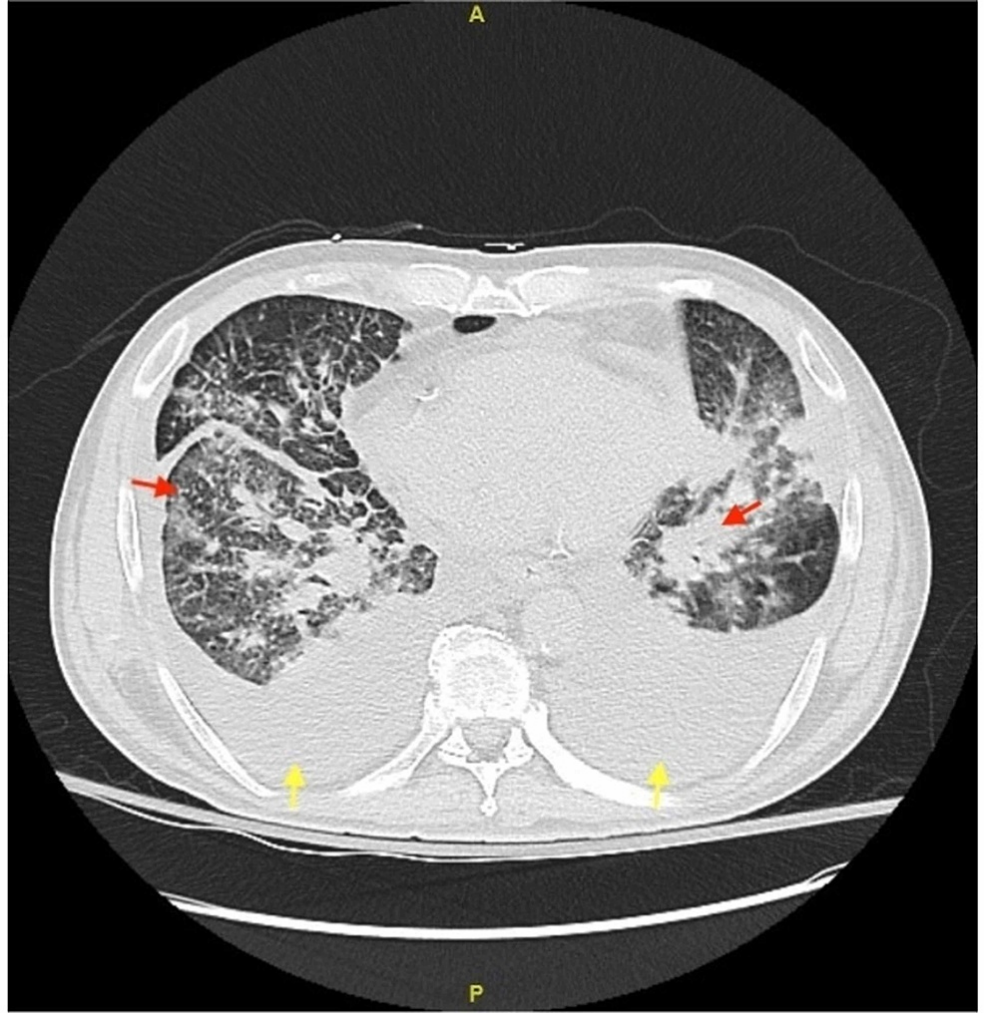
Computerized tomography of the chest. Bilateral pleural effusions (yellow arrows) with consolidation and possible metastases (red arrows).

On this admission, his initial vital signs were temperature 98.2 F, pulse 84 beats per minute, respiratory rate 15 breaths per minute, blood pressure 111/69 mmHg, and oxygen saturation 93% on room air. Physical examination was significant for rhonchi and lymphadenopathy. Chest X-ray and CT of the chest showed pneumonic infiltrates and pleural effusion (Figure 1 and Figure 2, respectively), which required drainage by thoracentesis.

As the patient had dysphagia and weight loss, a barium swallow was done, which was remarkable for slow emptying and severe backflow in the esophagus followed by coughing associated with dysphagia. The patient had undergone an esophagogastroduodenoscopy (EGD) due to findings on barium study. EGD revealed a gastric fundi mass approaching the gastroesophageal junction. Biopsies taken from the gastric-mass were positive for gastric adenocarcinoma. A repeat thoracentesis was performed. Pleural fluid cytology was positive for gastric adenocarcinoma.

**Figure 3:**
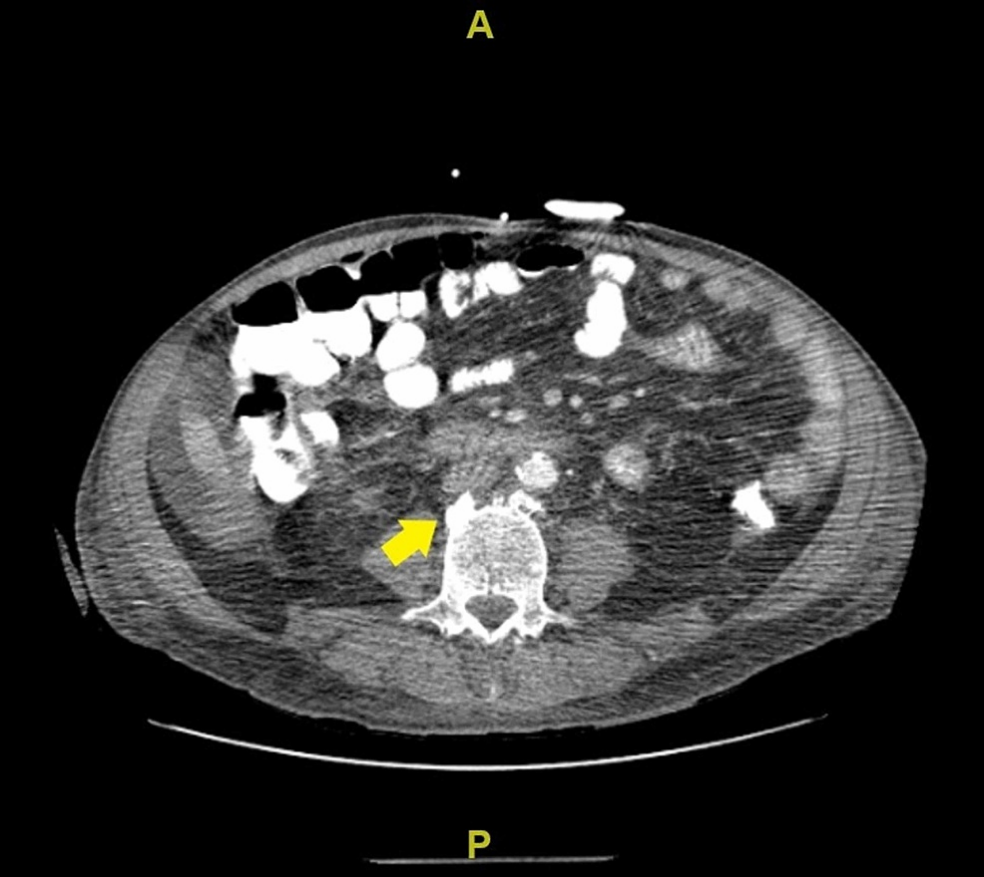
Computerized tomography scan of abdomen and pelvis. Sclerotic lesions in the thoracolumbar spine (yellow arrow) highly suspicious for osseous metastasis.

**Figure 4:**
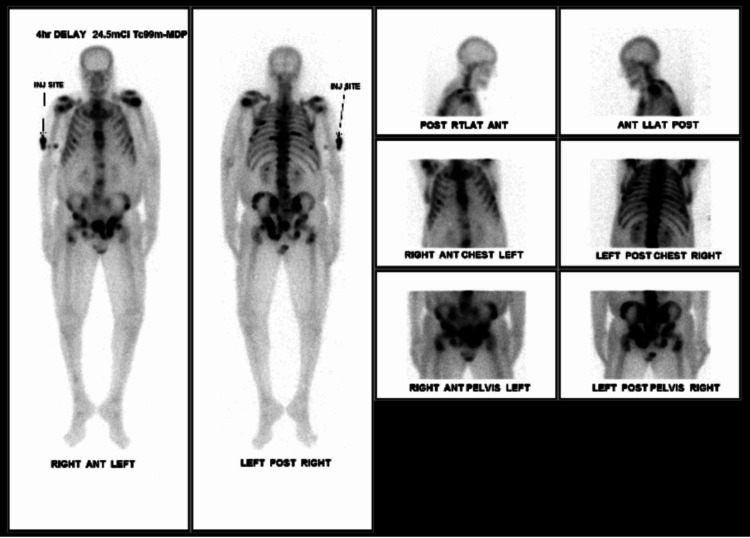
Bone scan. Multiple areas of increased uptake are seen involving the spine, ribs, pelvis and proximal femurs and left proximal humerus consistent with osseous metastatic disease.

A follow-up CT of the abdomen and pelvis and bone scan revealed metastatic disease (Figure 3 and Figure 4, respectively). Furthermore, the patient required bilateral indwelling pleural catheters secondary to having persistent pleural effusions and a percutaneous endoscopic gastrostomy tube for nutritional support. Oncology had determined that the patient was not a candidate for chemotherapy. Ultimately, the patient was transferred to an inpatient hospice unit as he had a short life expectancy due to having severe metastatic gastric adenocarcinoma.

## Discussion

Gastric cancer usually is diagnosed in the 6th decade of life, with an average of 68 years old [1,3-6]. However, our patient presented almost a decade earlier, likely due to multiple risk factors (male gender, smoking history, ethnicity, alcohol use). Early signs and symptoms of gastric cancer can be vague, more commonly attributing it to abdominal pain, weight loss, and nausea [1-4,8,9].

Smoking and heavy alcohol consumption increase the risk of gastric cancer by approximately 80% [1,7]. Our patient’s risk factors included smoking history, male gender, and ethnicity. On the other hand, a history of H. pylori, a Class I bacterial carcinogen that causes 90% of all gastric carcinomas, is a risk factor [1-5], which our patient did not have. 

The most common sites of metastases include the liver (48%) and peritoneum (32%) [10,11]. Less commonly, bone (12%), lung (15%), pleura/mediastinum (6%), or nervous system (3%) metastases can occur [10,11]. Our patient had cervical and axillary lymphadenopathy suggestive of lymph node metastases. Our patient also had distant metastases to the lung, pleura, and thoracolumbar spine.

Specifically, malignant pleural effusion is most commonly associated with lung cancer (35%-46%) and breast cancer (8%-40%) [12]. Malignant pleural effusions from gastrointestinal adenocarcinoma has a prevalence of 2% [12]. Additionally, cytology has a mean sensitivity of 60% [12,13], which may explain why the patient's initial cytology was negative. Although cytology is an approved initial test, it is dependent on cytologist experience, sample preparation, and primary tumor etiology [12].

Our patient’s prognosis remained poor due to having diffuse metastases. Unfortunately, metastatic gastric cancer has a poor prognosis with a five-year survival rate of 5% [14]. Treatment primarily consists of chemotherapy and palliative care [14]. This case report further illustrates that most cases of gastric cancer by the time of diagnosis have progressed to advanced, incurable disease [1,3,4].

## Conclusions

Although gastric cancer is one of the most common cancers worldwide, gastric cancer is still under-diagnosed in North America. Thorough history taking along with a strong clinical suspicion can improve gastric cancer morbidity and mortality. Also, it is important to coordinate care among multiple specialists as early as possible. These steps are vital to improving patient mortality, as well as quality of life.
